# Expression profile of small RNAs in *Acacia mangium* secondary xylem tissue with contrasting lignin content - potential regulatory sequences in monolignol biosynthetic pathway

**DOI:** 10.1186/1471-2164-12-S3-S13

**Published:** 2011-11-30

**Authors:** Seong Siang Ong, Ratnam Wickneswari

**Affiliations:** 1 School of Environmental and Natural Resource Sciences, Faculty of Science and Technology, Universiti Kebangsaan, Malaysia

## Abstract

**Background:**

Lignin, after cellulose, is the second most abundant biopolymer accounting for approximately 15-35% of the dry weight of wood. As an important component during wood formation, lignin is indispensable for plant structure and defense. However, it is an undesirable component in the pulp and paper industry. Removal of lignin from cellulose is costly and environmentally hazardous process. Tremendous efforts have been devoted to understand the role of enzymes and genes in controlling the amount and composition of lignin to be deposited in the cell wall. However, studies on the impact of downregulation and overexpression of monolignol biosynthesis genes in model species on lignin content, plant fitness and viability have been inconsistent. Recently, non-coding RNAs have been discovered to play an important role in regulating the entire monolignol biosynthesis pathway. As small RNAs have critical functions in various biological process during wood formation, small RNA profiling is an important tool for the identification of complete set of differentially expressed small RNAs between low lignin and high lignin secondary xylem.

**Results:**

In line with this, we have generated two small RNAs libraries from samples with contrasting lignin content using Illumina GAII sequencer. About 10 million sequence reads were obtained in secondary xylem of Am48 with high lignin content (41%) and a corresponding 14 million sequence reads were obtained in secondary xylem of Am54 with low lignin content (21%). Our results suggested that *A. mangium* small RNAs are composed of a set of 12 highly conserved miRNAs families found in plant miRNAs database, 82 novel miRNAs and a large proportion of non-conserved small RNAs with low expression levels. The predicted target genes of those differentially expressed conserved and non-conserved miRNAs include transcription factors associated with regulation of the lignin biosynthetic pathway genes. Some of these small RNAs play an important role in epigenetic silencing. Differential expression of the small RNAs between secondary xylem tissues with contrasting lignin content suggests that a cascade of miRNAs play an interconnected role in regulating the lignin biosynthetic pathway in *Acacia* species.

**Conclusions:**

Our study critically demonstrated the roles of small RNAs during secondary wall formation. Comparison of the expression pattern of small RNAs between secondary xylem tissues with contrasting lignin content strongly indicated that small RNAs play a key regulatory role during lignin biosynthesis. Our analyses suggest an evolutionary mechanism for miRNA targets on the basis of the length of their 5’ and 3’ UTRs and their cellular roles. The results obtained can be used to better understand the roles of small RNAs during lignin biosynthesis and for the development of gene constructs for silencing of specific genes involved in monolignol biosynthesis with minimal effect on plant fitness and viability. For the first time, small RNAs were proven to play an important regulatory role during lignin biosynthesis in *A. mangium*.

## Background

The spectacular escalation in complexity in the plant genomes correlates well with the aberrant increase in the number of naturally occurring small RNAs, termed as microRNAs (miRNAs) and small interfering RNAs (siRNAs). MicroRNAs are an abundant class of 19-24 nucleotides which are small endogenous non-coding RNA that negatively regulate gene expression at the post-transcriptional level by directing the cleavage of mRNAs or interfering with translation [1-5]. As key regulators of diverse biological processes, this group of small RNAs act by base pairing to complementary target sites and mediating mRNA cleavage or translation repression [1]. In addition, expression of these mature miRNAs varies between tissues and over time [6].

Biogenesis of miRNA occurs in the nucleus [7]. First, miRNAs are single stranded RNA molecules encoded by nuclear genes which are processed into primary miRNA (pri-miRNA) transcripts by the action of RNA polymerase II [7]. Then, these pri-miRNA are cropped into stem loop structures called precursor miRNAs (pre-miRNAs) with sequence of 70-150nt long through the action of RNase III [6,7]. Pre-miRNAs are then transported into the cytoplasm through the nuclear transport receptor complex [6,7]. Inside the cytoplasm, these pre-miRNAs with characteristics of stem loop secondary structures are further processed to generate mature miRNAs [7-9]. On the other hand, siRNAs are processed from long double stranded RNA (dsRNA) introduced exogenously into cells, formed by convergent transcription, extended hairpin structures or RNA-dependent RNA polymerization [7,8].

RNA interference (RNAi) is a natural phenomenon of specific gene silencing in fungi, plants and animals [7]. As RNAi has become a standard experimental tool in biological research, understanding their biogenesis is imperative in order to better understand the mechanism of gene silencing trigger by miRNAs and siRNAs [7]. In plants, RNAi machinery are triggered by the assembling of these small RNAs into RNA-induced silencing complexes (RISC) [7,10] which further guide the post-transcriptional gene silencing [11-15]. Although biogenesis of both mature miRNAs and siRNAs are different from each other, both of them depend on the Dicer for appropriate processing [16].

Although the mechanisms of RNAi initiated by miRNAs are about the same as siRNAs, however, miRNAs are said to play an essential role in plant growth, development and stress response [17,18]. MicroRNA play a critical regulatory behaviour in root, leaf, flower and shoot development [18-22]. Recent findings have demonstrated that majority of the plant miRNAs are conserved. For instance, miR156, miR159, miR164 and miR172 regulate the LFY expression, flowering time and floral organ identity which are important characteristics in the normal plant growth and development [23-26]. MiR413 regulates the expression of FLOWERING LOCUS C (FLC) [23]. Gene expression study has revealed that majority of the plant miRNA families in *Populus trichocarpa* are expressed at some level associated with cambium differentiation activities [17]. Tissues specific expression of miRNAs (ptr-miR160, 164, 171, 473, 477, 478, 479, and 480) suggested the specific roles of these miRNAs families in xylem tissue [17].

In *Arabidopsis*, bioinformatic analysis revealed that highly conserved miRNA families encoded transcriptional factor as their predicted target [11]. Several studies using model plant species have validated the roles of miRNAs in regulating the specific lignin pathway genes or the entire lignin biosynthetic pathway genes by controlling the binding between transcription factors with the AC elements present in the promoter of monolignol biosynthetic genes. Transcriptional factors like MYB, NAC, HD-Zip, SQUAMOSA PROMOTER BINDING PROTEIN-LIKE and many others are known to be differentially regulated in developing wood, tension wood, xylem, wood forming stem, developing stem and differentiating xylem [27-30]. Although conserved miRNAs families are present in all plant families, some of the isoforms might play a specific role with their expression being unique to that particular tissue of the species [17,31-34].

High throughput sequencing approach is so far the most economic and accurate method for miRNA isolation compared to computational and cloning based approach [35,36]. This is because cloning approach might exclude underexpressed miRNAs in a particular investigated tissue [35,36]. This limitation could be overcome using deep sequencing strategies as the entire low abundance novel small RNA classes could be discovered [35,36]. Identification of the entire set of small RNAs is the best strategy to better understand the mechanism of gene regulation and gene silencing in a complex organism [35-39]. In our study, Illumina GAII sequencer was employed to profile the relative expression of the small RNAs between samples with contrasting lignin content. Changes in the relative abundance of the expressed small RNAs between *A. mangium* secondary xylem with contrasting lignin content indicated the specific adaptation and behavior of the identified miRNAs or siRNAs.

## Results

### Lignin content characterization

Genotype differences are important for the variation implicated in the anatomical parameters of wood investigated. In secondary wall formation, variations in the lignin content are attributed by the adaptative behavior of the cambial elements [40]. Preliminary investigations showed that *A. mangium* and *A. auriculiformis* trees with big diameter at breast height (DBH) have lower lignin content than trees with small DBH. In this study, two *A. mangium* trees with difference in DBH were selected to determine their small RNA profiles. Klason lignin in Am48 (DBH = 35.5 cm) and Am54 (DBH = 58.0 cm) was 41% and 21% respectively. Both trees were 10 years old. As demonstrated in transgenic aspen and poplar (*Populus tremula* × *Populus alba*), lignin reduction are accompanied with higher cellulose content [41] together with substantially enhanced growth rate [42]. This finding suggested that wood with higher lignin content may repress the growth rate of trees as majority of the energy required during secondary cell wall formation are being used up extensively in lignin synthesis.

### Overview of small RNA profiles in samples with contrasting lignin content

Our goal was to identify miRNAs from secondary xylem that could be involved in the regulation of the lignin biosynthetic pathway in *A. mangium* species. We therefore used deep sequencing approach in order to capture the complete set of *A. mangium* small RNAs present. To this end, two small RNA libraries from secondary xylem tissue with contrasting lignin content were constructed using Illumina GAII high-throughput sequencing technology. Total RNA was size fractionated, ligated to 5’ and 3’ adapters, purified, PCR-amplified and sequenced using Illumina GAII sequencer. After adapter trimming, sequencing yielded 14 million reads in low lignin Am54 library while 10 million reads were obtained in high lignin Am48 library.

**Figure 1:**
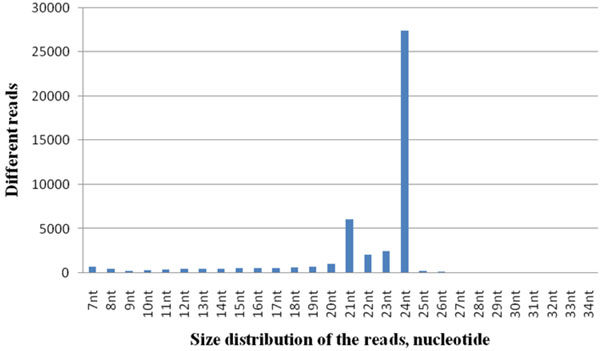
Total number of different reads in each class of sequences with a particular read length in Am48 and Am54.

We first examined the correlation between proportions of unique sequence signature or the total sequence reads with the length of small RNAs (Figure 1). Similar distribution was obtained between low lignin and high lignin libraries. Two major peaks at 21 and 24 nucleotides in the total sequences reads from contrasting lignin samples were obtained. Bioinformatics pipeline analysis revealed that majority of these sequences has roles in epigenetic silencing, metabolic process and transcriptional regulation. The 24-nucleotides small RNAs were dominant in their unique sequences and total reads, indicating high sequence diversity.

### Identification of 12 highly conserved miRNAs families

We focused on the 19- to 24- nt small RNAs for further analysis. BLAST analysis against the Genbank (http://www.ncbi.nlm.nih.gov/blast/Blast.cgi) showed that 12 highly conserved miRNA families were identified from *A. mangium* small RNA library with contrasting lignin content (Table 1). Further analysis against the miR Base (http://microrna.sanger.ac.uk/) and ASRP (http://asrp.cgrb.oregonstate.edu/) revealed that majority of these sequences have mismatches of not more than three nucleotides. These were classified as isoforms of the corresponding miRNAs belonging to a particular miRNA family (Additional file 1).

**Table 1:** Total counts of the 19-23 nt miRNAs of the 12 families isolated from secondary xylem tissues of Am54 and Am48. The 12 miRNA families shown are among highly conserved families in all plant species.

miRNA family	Am54	Am48
amg-miR159	3158	1008
amg-miR156	5340	1559
amg-miR166	1578	464
amg-miR164	5444	5371
amg-miR168	78825	33365
amg-miR172	32449	18189
amg-miR394	2037	226
amg-miR396	638	288
amg-miR160	29	32
amg-miR167	267	325
amg-miR162	990	175
amg-miR403	34	20

Our sequence analysis suggested that such highly conserved miRNAs might play an important role in the physiological adaptation of trees and wood formation in *A. mangium*. Difference in the relative expression of each of the individual isoforms between samples with contrasting lignin content implicated the roles of miRNAs in the secondary cell wall formation (Additional file 1). Majority of the predicted targets for these 12 *A. mangium* miRNA families were transcription factors which have been demonstrated to be highly associated with plant miRNA functions, playing a possible role in monolignol biosynthesis. This observation implicated the involvement of miRNAs network in specialized plant process such as wood formation in trees [17].

### *Acacia mangium* putative miRNAs and siRNA that are absent from *A. thaliana* and *P. trichocarpa*

We identified many new putative miRNAs and siRNA based on their expression level (Table 2). These novel putative miRNAs and siRNAs are not the degradation product as total RNA with RIN value >7 was selected for library construction. Furthermore, the expression level of these novel small RNAs between samples with contrasting lignin content suggests the presence of strong promoters in *A. mangium* genome to drive their expression. Taken together, the total numbers of predicted new putative miRNAs identified in this study are 82, which have strong differences in the expression level between Am48 and Am54 (Additional file 2). Furthermore, their expression levels are in 1,000 counts and some of these putative miRNAs have expression levels >10,000 counts. These newly identified putative miRNAs are conserved between samples with contrasting lignin content. MiRNAs sequences were distinguished from other small regulatory RNAs based on the ability to fold into hairpin miRNA precursors [43]. This criterion is generally accepted as evidence for the existence of a miRNAs [44]. As small interfering RNAs (siRNAs) are derived from the processing of long double stranded RNAs and are often of exogenous origin [16], we concluded that those 82 novel small RNAs are putative miRNAs specific to *A. mangium* genome based on the difference in their relative expression level between samples of contrasting lignin content.

**Table 2:** Counts of the 21-24 nucleotides non-conserved miRNAs isolated from secondary xylem tissues of Am54 and Am48 and their potential targets predicted to be involved in lignin biosynthetic pathway.

Sequence ID	Sequences (5’ ➙ 3’)	Counts Am54	Counts Am48	Target genes
15212(24)	AGAUGGUGAGGGAUCCAGGACUAU	17	18	*Populus trichocarpa* F-box family protein
13039(24)	UGGACGUACUGAACAACAAUGAAG	25	20	*Populus trichocarpa* F-box family protein
278(21)	UAAUCUGCAUCCUGAGGUAUA	281	286	*Populus trichocarpa* F-box
1045(24)	GAGGACCGAGAUCAUAGAUGAAGA	121	104	*Arabidopsis thaliana* peroxidase, putative
666(21)	UUCAUUGUCUGUUCGGCCCUG	67	121	*Zea mays* typical P-type R2R3 Myb protein
1391(21)	UUUGGCAUUCUGUCCACCUCC	522	57	*Populus trichocarpa* f-box family protein, mRNA
2100(21)	AAACGGGGUUGUGGGAGAGCA	15	36	*Populus* EST from mild drought-stressed leaves
2511(21)	GAGCUCAUCUUAGGACACCUG	46	29	*Populus* EST from mild drought-stressed leaves
3079(211)	AAGGUCGGCCAGUGAGACGAU	16	23	*Populus* EST from mild drought-stressed leaves
4169(21)	GACUACAAUUCGGACGCCGGG	22	16	*Populus* EST from mild drought-stressed leaves
5876(21)	ACGAUACUGUAGGGGAGGUCC	13	10	*Populus* EST from mild drought-stressed leaves
6077(21)	AGCGUAGAUCCGGAGAUUCCC	16	10	*Populus* EST from mild drought-stressed leaves
166(22)	GCCGGCCGGGGGAGGGACUGGG	150	127	*Populus* EST from mild drought-stressed leaves
297(22)	GCCGUCCGGGGGACGGACUGGG	194	72	*Populus* EST from mild drought-stressed leaves
1182(22)	GCCGGCCGGGGGACGGACUGCG	104	20	*Populus* EST from mild drought-stressed leaves
80(23)	GAUGGAACAAUGUAGGCAAGGGA	89	158	*Populus* EST from mild drought-stressed leaves

BLASTN search revealed that majority of the small RNAs obtained in this study did not have a match belonging to miRNA gene families in *Arabidopsis* or from different plant species. Some of these small RNAs have complementary sites upstream of the genomic DNA of *Oryza sativa*, *P. trichocarpa* and *A. thaliana* drought stress related genes and represent fragments of abundant non-coding RNAs (rRNA, tRNA and small nuclear RNA). We are not able to further differentiate these small RNAs with expression level >100 counts and <1,000 counts into putative miRNAs or siRNAs families as *A. mangium* genome sequences are not available yet. Majority of the remaining small RNAs obtained in this study can be classified as siRNAs as their expression level were <100 counts and some even had 10 counts only.

### Conserved miRNA families and isoforms

**Figure 2:**
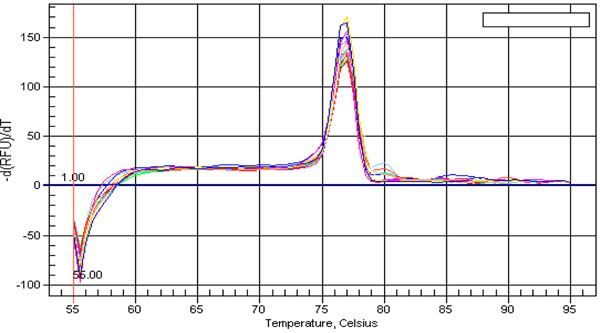
Dissociation curve of amplicon generated using mature miRNA sequences during quantitative real time – PCR.

miRNA sequences with a difference of less than three nucleotides were grouped into the same family and considered as isoforms. We further validated the selected conserved miRNAs families against miR Base and ASRP database. Interestingly, some of these families contained more than 10 isoforms. The expression level of certain isoforms was higher compared to other isoforms in secondary xylem tissues although they belong to the same family (Additional file 1). These differences in expression of the specific miRNA isoforms could be due to the spatial organization of these various isoforms in the genome. In addition, specific miRNA isoforms obtained corresponded to the registered sequences of other model plant species in the database. Conservation of particular miRNA isoforms across certain species suggests that some important aspects of their regulation may also be evolutionarily conserved [43]. This is proven based on the dissociation curve generated during real time – PCR to verify amplification specificity (Figure 2).

The relative expression of all the isoforms of the conserved miRNAs families in *A. mangium* are presented in Additional file 1. A total of four different isoforms belonging to amg-miR156 and three different isoforms belonging to amg-miR159 were discovered. Different isoforms belonging to a particular miRNA family exhibited different expression level in both samples with contrasting lignin content. For instance, isoforms with sequence ID 82(21) recorded 3605 counts in Am54 and a corresponding 1001 counts in Am48 (Additional file 1). This indicated the existence of a promoter upstream of these specific miRNA isoforms to drive their differential expression level in Am48 and Am54. As pri-miRNAs are located in the introns of host genes, this primary transcript might therefore be transcriptionally regulated through their host-gene promoters [2,5]. Furthermore, miRNAs which are clustered in polycistronic transcripts could also be coordinately regulated [2,5].

However, not much difference in the relative expression of all the isoforms belonging to amg-miR164 family between Am48 and Am54 was detected (Additional file 1). Out of these eleven different isoforms, isoform with sequence ID 13(21) showed highest expression with 5141 counts in Am54 and 5055 counts in Am48. The remaining isoforms belonging to these families were lowly expressed and showed slight differences in their relative expression in both samples.

### Prediction of gene targets for conserved and new amg-miRNAs

Putative targets of identified miRNAs were predicted using bioinformatics pipeline. The target genes of these conserved and novel small RNAs families were predicted. Among the targets identified, transcription factors are the predominant targets of the identified miRNAs. Transcription factors, including MYB transcription factor, SBP transcription factor, NAC transcription factor, HD-Zip transcription factor, APETALA 2-like transcription factor and Growth Regulating Factor (GRF) transcription factor were predicted to be the potential targets of *A. mangium* miRNAs. Others include stress related genes, peroxidase and F-box protein genes. Plant miRNAs regulate their target by having perfect and near perfect complementary sites with their target [11].

Other studies have shown that conserved miRNAs play extremely important roles in plant growth and development [31]. In plants, miR156 plays an important role by regulating the Squamosa-promoter binding protein (SPL) which is involved in the leaf development and their aberrant expression disrupt normal leaf and flower development. Our studies have shown that there is a difference in the expression pattern of this miRNA family in samples with contrasting lignin content which indirectly implies the diverse roles of this miRNA (Table 1). Hence, differences in the expression patterns of these miRNAs appear to be related to their function.

Based on the sequencing results obtained using deep sequencing approach, novel *A. mangium* small RNAs which play a specific role during developmental stages were also detected. These putative miRNAs and siRNAs displayed lower expression level compared to the majority of other conserved miRNA families. These novel small RNAs were detected in both samples with contrasting lignin content. We are not able to identify their homologues as the *A. mangium* genome sequence is not available yet.

Development- and tissue- specific expression of small RNAs are related to their physiological function [35]. Expression of small RNAs in different tissues, cell types and at different developmental stages varies [45-47]. Small RNAs present in the developing wood obtained in these studies indicated their specific roles during secondary wall formation.

### Transcriptional regulation of the lignin biosynthetic pathway by miRNAs

In *Arabidopsis*, reverse genetic analysis revealed by transcriptional factors has further expanded our understanding on the role of transcriptional regulation during wood formation [48-51]. In line with this, several classes of transcription factors derived from developing wood, tension wood, xylem, differentiating xylem, developing stem, wood forming stem and mature stem and hypocotyl stem have been identified in *Populus* sp, Loblolly pine, *Eucalyptus* sp., *A. thaliana* and *Zinnia elegans*[30]. In our work, several miRNA families with predicted gene targets encoding transcription factors were successfully discovered using *Arabidopsis* and *P. trichocarpa* genome reference sequences. We found that isoforms belonging to a particular family play an important role in regulating the lignin biosynthesis pathway genes.

**Figure 3:**
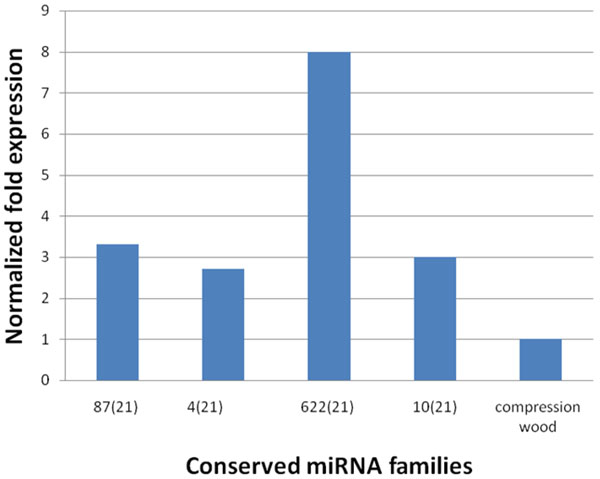
Relative normalized expression of four selected conserved miRNAs in compression wood (cw) and tension wood (tw) of *A. mangium*. 5.8S rRNA was used as a reference gene. The data are mean of three different individuals.

These analyses have uncovered 12 highly conserved miRNAs families whose relative expression changes significantly between Am48 and Am54. Further analysis using bioinformatics approach suggests that a cascade of miRNAs are involved in the regulation of lignin biosynthetic pathway genes in *A. mangium*. Out of the 12 highly conserved miRNA families identified, only four specific miRNAs were experimentally validated using quantitative real time PCR. Up-regulation of these miRNAs in xylem stress tension wood, a region enriched with cellulose content suggests their roles as a potential regulator in lignin biosynthetic pathway (Figure 3). This regulatory network involved a cascade of miRNAs. This is consistent with the validation in *A*. *thaliana*, whereby a battery of SND1-regulated transcriptional factors is required for normal secondary wall biosynthesis [52]. On the other hand, miRNA families like amg-miR396, amg-miR160 and amg-miR167 with transcription factor targets might play a less important role in regulating the machinery of lignin biosynthetic pathway in *A. mangium* (Additional file 1).

Although the expression of genes involved in the lignin biosynthetic pathway are highly coordinated, it is undeniable that different transcription factors might regulate the differing expression of the pathway genes [30]. Transcriptional profiling has been employed in many plant species to verify the preferential expression of many genes encoding transcriptional factors during wood formation [30]. A conserved miRNA family will display a unique role in different individuals due to the complexity in their regulatory network. Although SND1-regulated transcription factors were shown to act through a network of downstream transcription factors in *Arabidopsis*[*52*], however our results suggest contradictory evidence as there is not much variation in the total expression of all the isoforms of miRNAs belonging to amg-miR164 family (Additional file 1).

## Discussion

### High throughput sequencing of small RNAs

We have characterized small RNAs profiles from two contrasting lignin content samples using deep sequencing approach. Analysis of these small RNA expression patterns between Am48 and Am54 revealed that miRNAs are involved in the regulation of lignin biosynthetic pathway. The expression levels of some of these miRNAs were substantially high in low lignin Am54 while their expression level was very low in high lignin Am48. Bioinformatic analysis of the conserved *A. mangium* miRNAs against *P. trichocarpa* and *Arabidopsis* miRNA sequences deposited in miR Base and ASRP revealed that majority of the targets of these miRNAs are transcription factor genes which play an important role in the regulation of lignin biosynthetic pathway.

Majority of the genes upregulated in xylem tissue play an important role in secondary wall formation and lignin biosynthesis while bark-up-regulated genes had diverse function [53]. Highly conserved miRNAs with differing expression level between samples of contrasting lignin content in *A. mangium* secondary xylem most likely imply the important role of conserved miRNAs during secondary cell wall formation and lignin biosynthesis. As lignin biosynthetic pathway is quite often similar among plant species, differential expression of the miRNAs between xylem tissue with contrasting lignin content suggests an important role of miRNA since most miRNAs are well conserved across all animal and plant species [4,5]. Deep sequencing of secondary xylem tissues with contrasting lignin content and sequence comparison with other miRNA families deposited in miR Base revealed 12 conserved miRNA families in *A. mangium*. This implies that *A. mangium* has a collection of unique miRNAs sequences with specific roles in plant structure and defence.

The deep sequencing approach allowed us to discover and analyze the expression pattern of all the small RNAs that would not have been discovered using cloning based approach. Detailed comparison in the expression profiles of small RNA from similar tissues of contrasting lignin content showed differences in term of tissue-type-specific small RNAs. The relative expression of the majority of the small RNAs in this work that were extremely low would have been difficult to obtain using cloning based method. Landgraf et al. [54] speculated that these small RNAs might be derived from dsRNA structures that accidentally enter the RNAi pathway and are further processed by Dicer enzyme to produce siRNA. In *Drosophila melanogaster*, Dicer 2 generates siRNA from dsRNA which are important for DNA methylation [55,56]. Furthermore, transposable elements also serve as an important source for siRNA production that is required for imprinting [57].

### Lignin biosynthesis during secondary wall formation

As the second most abundant component in wood, lignin represent a major obstacle in the processing of wood for the pulp and paper industry. High lignin levels could limit the availability of carbon in cell division and growth [58]. This is because higher consumption of carbohydrate is needed during lignin synthesis which may have a negative impact on growth rate [58]. Evidence from several model plant species on the effect of low lignin on the growth of aspen and loblolly pine has been well established [42,58-60]. Similarly in this study, Am54 with a larger DBH (58.0cm) had low lignin (21%) whereas Am48 with smaller DBH (35.5cm) had high lignin (41%). Therefore, amount of lignin may directly influence the growth of *A. mangium*.

Wood formation involves a series of events beginning with cell division, lignifications until cell attenuation which lead to the formation of heartwood tissue [61]. Therefore, activity of the cambial elements will result in the formation of compression wood and tension wood known as reaction wood [62]. Reaction wood formation is induced in response to gravitational stimuli in nonvertically growing branch or stem [62-66]. In gymnosperms, its formation is induced in the lower side of the stem and known as compression wood [62-65]. Reaction wood in angiosperms is known as tension wood which has almost pure crystalline cellulose with decreased lignin content and is formed on the upper side of a branch [62-65]. The formation of this defective wood result in lower lignin content in tension wood and a corresponding higher lignin content in compression wood [62,64-66].

Gene expression studies in mutants with altered lignifications pattern have shown that variation in these traits are often attributed to other mechanism than variation in the pathway genes [67]. Furthermore, there are no well established scientific experiments on how the mechanisms of cambial element differentiation into specialized cells will either recruit a concomitant biosynthesis of preferred lignin types or transcriptional factors to form xylem or wood [67]. In line with this and based on the data generated using Solexa Second Generation Sequencing Technology, we believe that better understanding of the transcriptional factors and their miRNA regulators are important and further validation using real time Polymerase Chain Reaction is required.

In the phenylpropanoid pathway, some of the genes encoding enzymes contain common motifs that are recognized by specific transcription factors such as MYBs [58,68-71]. Transcription factor has the ability to bind to the promoter of lignin pathway genes to regulate their transcription [69,72-74]. Overexpression of the transcription factors will increase the thickness of the secondary cell wall and hence, alter the lignin profile [30]. As evidence, overexpression of the specific NAC transcription suppressed secondary wall deposition in the xylary fiber while increasing the wall thickness in the xylem vessels [75]. Among all the different classes of transcription factors implicated in lignification, MYBs are so far the most investigated candidates in the regulation of lignin biosynthetic pathway genes. The involvement of transcription factors in the transcriptional regulation of phenylpropanoid and flavanoid metabolic pathways has been well documented in a plant model system [76].

Secondary cell wall formation involves complex coordination of various pathway genes. In the cytoplasm, monolignols are synthesized and are exported into the cell wall to be polymerized giving rise to different lignin units [77]. Here, polymerization takes place extensively through the action of laccases and peroxidases [78,79]. Downregulation of these polymerizing enzymes are accompanied with altered lignin profile in the cell wall [78,79]. For instance, suppression of the laccase gene through antisense strategy in *Populus tremula* x *Populus alba* was accompanied with 2- to 3- fold increase in total soluble phenolics content (known as extractives) [79]. This implies that downregulation of polymerization enzymes has dramatic effects on the xylem fiber cell walls and indirectly indicates the involvement of laccase in xylem development [79].

Conserved miRNA families miR397 and miR408 with gene target for laccases and plastocyanin-like proteins that mediate lignin polymerization were not detected in this study. Instead, miRNAs with sequence ID 1045(24) with predicted gene target for peroxidase enzymes in *A. thaliana* was discovered. Laccase and peroxidase play an important role in monomer polymerization giving rise to lignin. Since the expression profile of small RNAs with sequence ID 1045(24) between both Am54 and Am48 is about similar, we postulated that lignin polymerization took place extensively in the sapwood tissues (Table 2). In vascular tissues, secondary wall thickening play a fundamental role in resisting the negative pressure generated in the xylem vessels due to transpiration and providing mechanical strength to support the plant body [75]. Hence, it is obvious that interactions of miRNAs with genes encoding transcription factors underlay regulation of the lignin biosynthetic pathway.

### Diverse roles of small RNAs associated with the formation of specialized wood

Mechanical stress can have profound effect on the spatiotemporal deposition of lignin, cellulose and hemicelluloses [17]. In the event the growth rate is fast, mother cell produced from the cambial initial may receive a further division before it could grow larger [40]. In fast growing trees, some cells may divide at a fast rate and can result in different degree of cell wall expansion and thickening [40,80]. The duration of cell division cycle in the cambial region will determine the rate of cell production [81]. Hence, mechanical stress result in different proportions of vessel elements as biosynthetic pathway of major woody cell wall components can be switched on and off. As an example, the numbers of vessels in tension wood are lower compared to compression wood [82-84].

Associations of miRNAs with cell or organ development in secondary cell wall formation suggest the involvement of a cascade of miRNAs during wood formation [17]. Based on the expression profiles of the small RNAs obtained in low lignin Am54 and high lignin Am48, upregulation and downregulation of certain small RNAs might affect the formation of various types of cells and organs which are important for plant development. A battery of miRNAs is implicated in regulation of various cells and organs required in wood development of the drastically upregulated and downregulated small RNAs in the low lignin Am54 and high lignin Am48.

Reference species such as *Arabidopsis* and *P. trichocarpa* can provide tremendous insights into *A. mangium* small RNA functions where the genome of this species is not sequenced and annotated yet. Comparative genomic analysis will allow us to identify the functions of identified small RNAs as majority of the pathway sequences are well conserved across all plant species. At the regulatory level, there might exist specific roles for conserved miRNAs and other small RNAs which are specific to a particular plant species. For example, high level of ptr-miR159 expression was detected in poplar stem but was weakly expressed in leaves [17]. However, in *Arabidopsis*, expression of this miRNAs was conspicuous in leaves and was not detectable in stems [33]. Expression of ptr-miR164 is significantly higher in *Populus* stem than in leaves but such expression was not detected in *Arabidopsis*[17,34]. In this study, no preferential expression of this amg-miR164 family was detected in sapwood of low and high lignin *A. mangium* trees (Table 1). The sequences generated in this study give immense contributions in understanding of the regulatory mechanism of small RNAs in lignin biosynthesis and secondary wall formation.

### Small RNAs with putative functions in epigenetic silencing

In eukaryotes, epigenetic regulation via transcriptional silencing involves methylation of cytosine residues [85]. In *Physcomitrella patens*, methylation of the promoter of gene encoding miRNA targets demonstrated specific epigenetic changes in their promoter region [56]. Induction of epigenetic silencing by DNA methylation in an organism depends on the miRNA and its gene target ratio [56]. If the concentrations of miRNA exceed a threshold, two mechanisms are employed in gene silencing. The miRNAs are loaded into the RNA Induced Transcriptional Silencing and also interact directly with their respective gene targets [56]. Analysis of null mutants of various DICER isoforms suggests that miRNA and DNA methylation are both way interaction [56]. We found that certain distinct small RNAs demonstrated expression level up to 170,000 counts and 121,000 counts in samples with contrasting lignin content while some small RNAs exhibited lower expression level. Wu et al. [86] reported that Argonaute 4 (AGO4)-associated 24 nt lmiRNA can direct methylation both at the locus from which it is produced and the target site. Our sequence ID 1(22) exhibited 131,834 counts in low lignin Am54 and 51,433 counts in high lignin Am48. Methylation related siRNA production requires complex coordination of various members from siRNA biogenesis and RNA transcription pathway. In this complex methylation machinery, AGO4 is one of the most important components [87,88]. Interestingly, this distinct expression level of small RNA, sequence ID 1(22), between contrasting lignin tissue suggests its role in epigenetic silencing through methylation of the CpG island or histone modification during secondary wall formation in wood.

In *Arabidopsis*, small RNA with 24nt length can direct chromatin remodeling and DNA methylation at their corresponding loci [89]. Reprogramming events take place in the genome of endosperm and pollen resulting in the production of distinct classes of small RNA [90]. In *Arabidopsis*, these small RNAs could influence the transposable element activity and chromosome behavior after fertilization [90]. Almost 80% of the small RNA sequences generated from the *A. mangium* libraries with contrasting lignin content belong to 24 nt lengths and their distribution pattern suggested roles in imprinting and reprogramming events. Recent progress in epigenetic differences indicated that small RNA has the potential in targeting machinery involved in DNA methylation and chromatin modifications [91,92]. As a result, association of imprinting and reprogramming events with the activity of transposable elements can influence expression of neighboring genes [93,94]. Epigenetic event is not only restricted to small RNA families with 24nt lengths but do involve small RNAs with 21nt lengths. In *A. thaliana*, the genome sequence provided an excellent resource to understand the epigenetic roles of miR165/166 [95]. They interact with the newly processed PHB mRNA to change the chromatin of the target gene template [95]. This implies that the five isoforms of miR166 family in *A. mangium* may assume a dual role i.e. RNAi and epigenetic silencing in controlling gene expression.

## Conclusions

Our study critically demonstrated the roles of small RNAs during secondary wall formation. Comparison of the expression patterns of small RNAs between secondary xylem tissues with contrasting lignin content strongly indicated that small RNAs play a key regulatory role during lignin biosynthesis. Our analyses suggest a evolutionary mechanism for miRNA targets on the basis of the length of their 5’ and 3’ UTRs and their cellular roles. The results obtained can be used to better understand the roles of small RNAs during lignin biosynthesis and for the development of gene constructs for silencing of specific genes involved in monolignol biosynthesis with minimal effect on plant fitness and viability. For the first time, small RNAs were proven to play an important regulatory role during lignin biosynthesis in *A. mangium*.

## Methods

### Plant materials

Plant materials for lignin content determination and small RNA analysis were taken from 10 years old *A. mangium* trees in Plot W, Plant Biotechnology Centre, UKM, Bangi, Malaysia. Two trees with distinctly different DBH (58.0cm and 35.5cm) were selected for lignin content determination. Sapwood tissues with contrasting lignin content were used in the small RNA analysis. A large Dewar containing liquid nitrogen was brought to the field on a small truck. Wood disc was sawed and immediately immersed in liquid nitrogen. Wood disc was chipped into about 1cm^3^ thick cookies using chisel and hammer, and washed with RNase Away solution (Invitrogen. Carlsbad, CA). The cookies were transferred into marked bags and immediately stored inside -80°C freezer until used.

### Determination of lignin content

Lignin content of sapwood tissues of 10 years old *A. mangium* was estimated using protocol described in TAPPI T 222-om-88. Klason lignin of the investigated tissues was determined based on the extractive free wood method. About 1 g of wood meal was placed in a 100mL beaker followed by the addition of 15mL of 72% H_2_SO_4_. Mixture was subjected to occasional stirring for 2 h at room temperature. Solution was transferred into a 1-L Erlenmeyer flask and topped up with deionized water until it reached 575mL and refluxed under heating for 4 h. The solution was filtered using crucible No. 4 and the acid insoluble lignin was determined gravimetrically.

### Small RNA isolation, library construction and Solexa sequencing

Total RNA was isolated from sapwood tissues with contrasting lignin content using miRVana miRNA Isolation Kit (Ambion, Austin, TX, USA) following manufacturer’s protocol. Thin cookies were first ground in a blender and then further ground to fine powder using mortar and pestle. Integrity of the isolated Total RNA was analyzed using RNA 6000 Nano kit (Agilent Bioanalyzer, USA) and only Total RNA with RIN value above 7 was selected for library construction. Low molecular weight RNA was purified using the Ambion Flash PAGE system following manufacturer’s instructions. For the library preparation, Illumina DGE small RNA Sample Preparation Kit Cat. No. FC-102-1009 was used following manufacturer’s instructions. Low molecular weight RNA was ligated with 5’ and 3’ adapters separately and gel purification of the adapter ligated cDNA was done. Two constructed cDNA libraries were used for sequencing using Illumina Solexa GA II.

### Bioinformatics pipeline analysis

Individual sequence reads with high quality scores were filtered from the raw data generated by Illumina Solexa GA II. After removal of adapter sequences, reads from 7 to 35 nucleotides were chosen. All identical sequences were counted and the resulting set of unique sequences with associated read counts were referred to as sequence tags. Clustering based on relative lengths was done using in house perl scripts. A total of 14,582,383 reads were generated in low lignin Am54 and 10,281,313 reads in high lignin Am48.

### Gene target prediction and validation

Sequences data were analyzed for their predicted gene target family using homology search against the database in NCBI [96]. Only sequences with mismatches of not more than 3nt were grouped to the particular miRNA family. Following this, the target genes of the identified miRNAs sequences were further validated using two public resources which were miR Base (http://microrna.sanger.ac.uk/) and ASRP (http://asrp.cgrb.oregonstate.edu/). miR Base provides a list of all the mature miRNAs and their precursor sequences in plant species [18,97]. We further compared all our 12 highly conserved mature miRNA sequences with the sequences available in miR Base database to further confirm the mature miRNA sequences and their predicted precursor structures. ASRP database was used to predict the gene targets of identified conserved miRNA families using *Arabidopsis* as a model plant [18,98]. The gene target sequence belonging to a particular miRNA family was downloaded from ASRP database and was used to search for binding energy between our highly conserved mature miRNA sequence using psRNA Target: A Plant Small RNA Target Analysis Server [99].

### miRNA expression validation in wood using quantitative real-time PCR

Four conserved miRNAs with preferential expression between low lignin Am54 and high lignin Am48 were experimentally validated across three different individuals of two years old *A. mangium* in Plot A, Plant Biotechnology Centre, UKM, Bangi, Malaysia. Validation of the selected miRNAs was conducted using quantitative real-time PCR in xylem stress compression wood and xylem stress tension wood. Total RNA was isolated from tension wood and compression wood using miRVana miRNA Isolation Kit (Ambion, Austin, TX, USA) and treated with RNase-free DNase 1 (Qiagen, Germany). Integrity of the isolated Total RNA was analyzed using RNA 6000 Nano kit (Agilent Bioanalyzer, USA) and only Total RNA with RIN value above 7 was used in the subsequent real time PCR experiment. Total RNA was reverse transcribed using miScript Reverse Transcription Kit (Qiagen, Germany) with the addition of poly (A) tails to the 3’ end of the RNA. Investigated miRNA was amplified from the reverse-transcribed cDNAs using mature miRNA sequence as the forward sequence (Table 3) and miScript Universal Primer as the reverse primer. 5.8S rRNA was selected as the endogenous reference gene. Real time PCR was performed using Bio-Rad iQ5 (USA). For each PCR, 1μl cDNA template equivalent to 100pg total RNA was mixed with 10μl of 2x QuantiTect SYBR Green PCR Master Mix (Qiagen, Germany), 2μl of 10x miScript Universal Primer (Qiagen, Germany) and 5pmol of the forward primers in a final volume of 20μl. Thermal denaturing step was performed to generate the dissociation curve in order to verify the amplification specificity. PCR amplification protocol was as follows: 15 min at 95°C for PCR initial activation step, 45 cycles consisting of 15 s at 94°C, 30 s at 55°C and 30 s at 70°C and final extension step of 4 min at 70°C.

**Table 3:** List of the miRNA primers used in quantitative real time PCR analysis in compression wood and tension wood of 2 years old *A. mangium.*

Sequence ID	Sequence ( 5’ ➙ 3’ )	miRNA family
87(21)	TTTGGATTGAAGGGAGCTCTA	amg-miR159

4(21)	TCGCTTGGTGCAGGTCGGGAA	amg-miR168

10(21)	AGAATCTTGATGATGCTGCAG	amg-miR172

622(21)	TTGGCATTCTGTCCACCTCCC	amg-miR394

## Competing interests

Both authors declare that they have no competing interests.

## Authors’ contributions

SSO performed the laboratory work in this study and organized the results. SSO and RW conceived this study and designed experiments. The first draft of this manuscript was written by SSO and revised by RW. Both authors read, corrected and approved the final manuscript.

## Supplementary Material

Additional file 1 **Conserved miRNAs families in *A. mangium* with corresponding isoforms**. The 12 highly conserved plant miRNA families with strong differences in the expression level in each of the isoforms between low lignin Am54 and high lignin Am48 Click here for file

Additional file 2 **Novel miRNAs identified from *A. mangium***. 82 novel putative miRNAs sequences identified from deep sequencing of *A. mangium* secondary xylem with very different expression levels in Am48 and Am54. Click here for file
