# Supplementary material for: Expression profile of small RNAs in Acacia mangium secondary xylem tissue with contrasting lignin content - potential regulatory sequences in monolignol biosynthetic pathway
Source: BMC Genomics. 2011 Nov 30;12(Suppl 3):S13. doi: 10.1186/1471-2164-12-S3-S13 (PMC3333172; doi:10.1186/1471-2164-12-S3-S13)
Supplement: Additional file 1 — Conserved miRNAs families in A. mangium with corresponding isoforms. The 12 highly conserved plant miRNA families with strong differences in the expression level in each of the isoforms between low lignin Am54 and high lignin Am48 [file 1471-2164-12-S3-S13-S1.pdf]

Additional file 1. Conserved miRNAs families in *A. mangium* with corresponding isoforms. The 12 highly conserved plant miRNA families with strong differences in the expression level in each of the isoforms between low lignin Am54 and high lignin Am48

miRNA Family	Sequence ID	miRNA Sequences (5' → 3')	Counts Am54	Counts Am48	Target
amg-miR156	3153(21)	UUGACAGAAGAGAGUGAGCAC	130	22	Squamosa promoter
	82(21)	UUGACAGAAGAUAGAGAGCAC	3605	1001	Binding Protein
	669(20)	UGACAGAAGAUAGAGAGCAC	48	17	(SBP) box
	23(20)	UGACAGAAGAGAGUGAGCAC	1557	519	
amg-miR159	966(20)	UUGGAUUGAAGGGAGCUCUA	97	11	MYB transcription
	87(21)	UUUGGAUUGAAGGGAGCUCUA	2967	958	factor
	214(19)	UUUGGAUUGAAGGGAGCUC	94	39	
amg-miR160	2293(21)	GCGUAUGAGGAGCCAAGCAUA	29	32	Auxin response transcription factors
amg-miR167	474(21)	UGAAGCUGCCAGCAUGAUCUA	105	175	
	82(22)	UGAAGCUGCCAGCAUGAUCUAA	82	73	
	364(22)	UGAAGCUGCCAGCAUGAUCUGA	53	61	Auxin response
	1465(22)	UGAAGCUGCCAGCAUGAUCUGG	27	16	transcription factors
amg-miR162	1637(21)	UCGAUAAACCUCUGCAUCCAA	297	48	DCL
	633(21)	UCGAUAAACCUCUGCAUCCAG	693	127	
amg-miR164	3264(21)	UGGCGAAGCAGGGCACGUGCA	11	21	
	5650(21)	UGGAGAAGCAGGGCACGUUCA	13	11	
	5855(21)	UGGAGAAGCAGGGUACGUGCA	11	10	NAC domain
	6042(21)	UGGGGAAGCAGGGCACGUGCA	10	10	transcription
	1962(21)	UGGAGAAGCAGGGCACGUGCU	46	39	factor
	13(21)	UGGAGAAGCAGGGCACGUGCA	5141	5055	
	589(21)	UGGAGAAGUAGGGCACGUGCA	105	134	
	674(20)	GGAGAAGCAGGGCACGUGCA	24	16	
	234(20)	UGGAGAAGCAGGGCACGUGC	57	51	
	5338(21)	UGGAGAAUCAGGGCACGUGCA	11	12	
	5356(21)	UGGAGAAGCAGGGCACGUGCC	15	12	
amg-miR166	256(21)	UCUCGGACCAGGCUUCAUUCC	984	324	
	2731(21)	UUUCGGACCAGGCUUCAUUCC	79	26	HD-Zip transcription
	2556(21)	CUCGGACCAGGCUUCAUUCCU	86	28	factor
	933(20)	UCUCGGACCAGGCUUCAUUC	22	11	
	1090(21)	UCGGACCAGGCUUCAUUCCCC	407	75	
amg-miR168	1254(21)	UCGCUUGGUGCAGGUCGGGAC	88	64	
	2219(21)	UCUCUUGGUGCAGGUCGGGAA	72	34	
	2015(21)	UCGCUUGGUUCAGGUCGGGAA	46	37	
	1955(21)	UCGCUUGGUGCAGGUCGGGCA	81	39	
	567(21)	UCGCUUGGUGCAGGUCGGGAU	394	139	Argonaute (AGO1)
	623(21)	UCGCUUGGUGCAGGGCGGGAA	75	128	
	828(21)	UUGGUGCAGGUCGGGAACCGG	288	96	
	953(21)	UCGCUUGGGGCAGGUCGGGAA	60	84	
	2988(21)	UCGCUUGGUGCAGUUCGGGAA	67	23	
	2482(21)	CGCUUGGUGCAGGUCGGGAAU	70	29	
	3375(21)	UCGCUUGGUGCAUGUCGGGAA	58	21	
	3691(21)	AUCGCUUGGUGCAGGUCGGGA	20	19	
	3(20)	GUGGCAUGUGUGGAACGGCA	2974	1723	
	4(20)	CGCUUGGUGCAGGUCGGGAA	2841	1053	
	1418(21)	CGCUUGGUGCAGGUCGGGAAC	210	56	
	2219(21)	UCUCUUGGUGCAGGUCGGGAA	72	34	
	3749(21)	UCGCUUGGUGCAGGUCGGCAA	33	18	
	3953(21)	UCGCUUGGUGCAGGACGGGAA	45	17	
	3965(21)	UCGCUUGGUGCAGGUCCGGAA	30	17	
	4077(21)	UCGCUUGGAGCAGGUCGGGAA	24	17	
	4675(21)	UCGCUUGGUGCCGGUCGGGAA	21	14	
	4(21)	UCGCUUGGUGCAGGUCGGGAA	71256	29703	

Additional file 1. (Continue)

Conserved miRNAs families in *A. mangium* with corresponding isoforms. The 12 highly conserved plant miRNA families with strong differences in the expression level in each of the isoforms between low lignin Am54 and high lignin Am48.

miRNA Family	Sequence ID	miRNA Sequences (5' → 3')	Counts Am54	Counts Am48	Target
amg-miR172	3681(21)	GGAAUCUUGAUGAUGCUGCAU	34	19	
	1788(21)	GUAGCAUCAUCAAGAUUCACA	73	43	
	1335(21)	GGAAUCUUGAUGAUGCUGCAC	196	60	
	2849(21)	AGAAUCUUGAUGAUGUUGCAG	42	25	
	2372(21)	AGAAUCUUGAUGAUGCUGCCU	94	31	
	2316(21)	AGAAUUUUGAUGAUGCUGCAU	73	32	
	3482(21)	AGAAUAUUGAUGAUGCUGCAU	15	20	
	3499(21)	AGCAUCUUGAUGAUGCUGCAU	25	20	
	3806(21)	AGAAUCUUGAUGAUGUUGCAU	74	18	
	3529(21)	AGAAUCUUGAUGAUGCUGCCG	52	20	APETALA2-like
	598(20)	GUAGCAUCAUCAAGAUUCAC	47	18	transcription factor
	227(20)	GAAUCUUGAUGAUGCUGCAU	75	53	
	226(19)	GGAGCAUCAUCAAGAUUCA	18	36	
	82(20)	AGAAUCUUGAUGAUGCUGCA	285	160	
	970(21)	AGAAUCUUGAUGAUGCUGCAC	99	83	
	2040(21)	GCAGCAUCAUCAAGAUUCACA	65	37	
	3108(21)	AGAAUCUUGAUGAUGCUGGAG	29	22	
	4816(21)	AGAAUCUUGAUGAUGCUGUAU	43	14	
	5757(21)	AGAAUCUUGAUGAUUCUGCAU	25	10	
	6043(21)	AGAAUCUUGAUGAUGAUGCAU	12	10	
	651(22)	AGAAUCUUGAUGAUGCUGCAGU	77	38	
	1008(22)	GGAGCAUCAUCAAGAUUCACAU	26	24	
	1719(22)	GAAUCUUGAUGAUGCUGCAUU	17	13	
	620(23)	AGAAUCUUGAUGAUGCUGCAGUA	48	33	
	10(21)	AGAAUCUUGAUGAUGCUGCAG	14505	8703	
	11(21)	AGAAUCUUGAUGAUGCUGCAU	16400	8647	
amg-miR394	1391(21)	UUUGGCAUUCUGUCCACCUCC	522	57	
	622(21)	UUGGCAUUCUGUCCACCUCCC	1157	129	F-box proteins
	297(20)	UUGGCAUUCUGUCCACCUCC	358	40	
amg-miR396	826(21)	GCUCAAGAAAGCUGUGGGAUA	191	97	Growth Regulating
	426(21)	GUUCAAGAAAGCUGUGGGAGG	447	191	Factor (GRF)
amg-miR403	3441(21)	CGAGUUUGUGCGUGAAUCUAC	34	20	Unknown
