# Supplementary material for: Expression profile of small RNAs in Acacia mangium secondary xylem tissue with contrasting lignin content - potential regulatory sequences in monolignol biosynthetic pathway
Source: BMC Genomics. 2011 Nov 30;12(Suppl 3):S13. doi: 10.1186/1471-2164-12-S3-S13 (PMC3333172; doi:10.1186/1471-2164-12-S3-S13)
Supplement: Additional file 2 — Novel miRNAs identified from A. mangium. 82 novel putative miRNAs sequences identified from deep sequencing of A. mangium secondary xylem with very different expression levels in Am48 and Am54. [file 1471-2164-12-S3-S13-S2.pdf]

Additional file 2. Novel miRNAs identified from *A. mangium*. 82 novel putative miRNAs sequences identified from deep sequencing of *A. mangium* secondary xylem with very different expression levels in Am48 and Am54.

Sequence ID	Sequence	Am54 (counts)	Am48 (counts)
1(19)	GGGGAUGUAGCUCAGAUGG	12824	6525
2(19)	GCUUGAAAUUGUCGGGAGG	2264	3442
3(19)	UGGGCUGGGUCGGUCGGUC	1278	2355
4(19)	GGGCUGGGUCGGUCGGUCC	2859	1510
14(19)	GCGCCUGUAGCUCAGUGGA	1758	453
35(19)	GGGGGACGGACUGGGUCGG	1153	216
104(19)	GUAAGGAUUGACAGACUGA	1421	77
3(20)	GUGGCAUGUGUGGAACGGCA	2974	1723
4(20)	CGCUUGGUGCAGGUCGGGAA	2841	1053
1(21)	GGAAUGGGCUGUUUGGGAAGA	121182	169567
2(21)	GUGGCAUGUGUGGAACGGCAC	126788	96834
3(21)	UAUGACUCAAGACGCACUGAU	33387	33817
5(21)	GGGGAUGUAGCUCAGAUGGUA	9754	16724
6(21)	GCCGGCCGGGGGACGGACUGG	25045	13468
7(21)	AUCCGGGCUAGAAGCGACGCA	9750	13133
8(21)	GGUGGCAUGUGUGGAACGGCA	14795	12663
9(21)	GUUAGAUCAAGAUUUGGACGG	14288	9683
10(21)	AGAAUCUUGAUGAUGCUGCAG	14505	8703
12(21)	UGGCAUGUGUGGAACGGCACC	8622	7952
14(21)	AAAUUGUUUGAAUCGUUGGGU	3599	4703
15(21)	CCAUGGGCUUGCAGAUGAGGG	9735	4701
16(21)	AGAAUCCGGGCUAGAAGCGAC	5376	4490
17(21)	GAAAUUGUUUGAAUCGUUGGG	3164	4443
19(21)	AAAGAAUGAGACGUGUAGCAC	4014	3673
20(21)	GAAUCUCAGUGGAUCGUGGCA	3118	3619
21(21)	AUGUGUGGAACGGCACCUUUG	4047	3521
22(21)	CCGGGCUAGAAGCGACGCAUG	1395	3376
23(21)	GAAUCGGAGCGACUUGGGCUG	3257	3081
24(21)	ACAGCAAUGAUUGUAGAGGCA	2469	2986
25(21)	UGAGACGGGUGAUGCAACUGA	7109	2817
26(21)	ACAAGCAGCUGAAAUUCUGAA	5100	2742
27(21)	AACCUCGAUCCGGACUAGGCA	1440	2732
29(21)	CCAAUGUGAAGGGGCUGGUCA	6851	2679
28(21)	GGAAUGGGCUGUUUUGGAAGA	3312	2683
30(21)	AUGGUUGAGACUAGGACGGUA	1884	2639
31(21)	CAUGUGUGGAACGGCACCUUU	2184	2500
34(21)	UGGUGCAGGUCGGGAACCGGU	4720	2462
35(21)	AAUUGUUUGAAUCGUUGGGUC	1778	2231
33(21)	UGGAGGAAUGUAGCCACAGCA	3528	2497
36(21)	GCAUGUGUGGAACGGCACCUU	3550	2212
38(21)	UCUCAGUGGAUCGUGGCAGCA	1562	2071
39(21)	GGGGAUGUAGCUCAAAUGGUA	1259	2069
41(21)	UGGACCCGAUGCUGACAGGAG	3565	1929
42(21)	GUUGGAUCAAGAUUUGGACGG	2444	1873
44(21)	ACAUUAAAAGGAGAUGAACAG	3629	1844
46(21)	UUUAGGAAGGCUUCGAACAGA	3042	1811
47(21)	CGUGAAUCGGAAGCGGGGCAU	2512	1783
48(21)	CAGCAAUGAUUGUAGAGGCAU	1436	1760
49(21)	AGCGGACUGCAGCGGAGGCAA	1043	1750
50(21)	GGUCUAUGAAGUGGAUGGUAG	2309	1629
54(21)	CUUUAGGAAGGCUUCGAACAG	3233	1527
55(21)	AGACGGAGGAAGUGUGAGUGA	3004	1527
56(21)	GGAAUGGGCUGUUUGGGGAGA	775	1497
64(21)	UGACUCAAGACGCACUGAUUG	3032	1368
65(21)	UUGAGCACGCUGUAUUACGCA	2191	1334
69(21)	UUUCGGACCAAUUUAAUGGCA	3696	1201
77(21)	UGAACGAAGGUACGAAGUCUA	2104	1084
80(21)	UGUGAAUGAUGCAGGAGCUAA	2049	1032
81(21)	UUUUCAAUUGGAGAAGGGCUU	2187	1006
83(21)	AGGAGACGUGUAGCACGUCGA	2287	995
87(21)	UUUGGAUUGAAGGGAGCUCUA	2967	958
109(21)	UCAGACAGUCUGACGUGACAU	2237	794
1(22)	GCCGGCCGGGGGACGGACUGGG	131834	51433
2(22)	GGGGAUGUAGCUCAGAUGGUAG	4280	8508

Additional file 2. (continued)
Novel miRNAs identified from *A. mangium*. 82 novel putative miRNAs sequences identified from deep sequencing of *A. mangium* secondary xylem with very different expression levels in Am48 and Am54.

Sequence ID	Sequence	Am54 (counts)	Am48 (counts)
3(22)	UCCGGGCUAGAAGCGACGCAUG	2291	7598
4(22)	GGAAUGGGCUGUUUGGGAAGAC	2474	3984
6(22)	CUUUAGGAAGGCUUCGAACAGA	4106	2220
8(22)	GCCGGCUGGGGGACGGACUGGG	6615	1889
17(22)	GCCGGUCGGGGGACGGACUGGG	3280	1091
24(22)	UCGCUUGGUGCAGGUCGGGAAC	2114	779
1(23)	CAACUGGCAAAGGGGUCGGACAC	5595	9528
2(23)	GGGGAUGUAGCUCAGAUGGUAGA	3915	7775
3(23)	AUCCGGGCUAGAAGCGACGCAUG	1555	4474
4(23)	GCCGGCCGGGGGACGGACUGGGA	9716	3388
5(23)	GGGGAUGUAGCUCAAAUGGUAGA	1151	2475
6(23)	GGUGAAGUGUUCGGAUCGCGGCG	6186	2342
26(23)	GCCGGCCGGGGGACGGACUGGGG	2201	532
1(24)	CAACUGGCAAAGGGGUCGGACACU	2295	4188
4(24)	CAAUGAGAGUUGGCAUAGAUGGUA	1937	3242
18(24)	CCAAGAGGGCGUAGCUGAGAUGGU	1782	1236
23(24)	GGUGAAGUGUUCGGAUCGCGGCGA	2885	1064
45(24)	CGAAUCCGGAUUAGUCGGCCCAGU	1462	747
